# Are all n-3 polyunsaturated fatty acids created equal?

**DOI:** 10.1186/1476-511X-8-33

**Published:** 2009-08-10

**Authors:** Breanne M Anderson, David WL Ma

**Affiliations:** 1 Department of Human Health and Nutritional Sciences, University of Guelph, Guelph, Ontario, N1G 2W1 Canada

## Abstract

N-3 Polyunsaturated fatty acids have been shown to have potential beneficial effects for chronic diseases including cancer, insulin resistance and cardiovascular disease. Eicosapentaenoic acid (EPA) and docosahexaenoic acid (DHA) in particular have been studied extensively, whereas substantive evidence for a biological role for the precursor, alpha-linolenic acid (ALA), is lacking. It is not enough to assume that ALA exerts effects through conversion to EPA and DHA, as the process is highly inefficient in humans. Thus, clarification of ALA's involvement in health and disease is essential, as it is the principle n-3 polyunsaturated fatty acid consumed in the North American diet and intakes of EPA and DHA are typically very low. There is evidence suggesting that ALA, EPA and DHA have specific and potentially independent effects on chronic disease. Therefore, this review will assess our current understanding of the differential effects of ALA, EPA and DHA on cancer, insulin resistance, and cardiovascular disease. Potential mechanisms of action will also be reviewed. Overall, a better understanding of the individual role for ALA, EPA and DHA is needed in order to make appropriate dietary recommendations regarding n-3 polyunsaturated fatty acid consumption.

## Introduction

In recent years, there has been increased focus on the role of specific dietary fatty acids and their effect on health and disease. N-3 polyunsaturated fatty acids (PUFA) have demonstrated a wide range of health-related benefits including improving heart disease related outcomes, decreasing tumour growth and metastasis, and favourably modifying insulin sensitivity. Eicosapentaenoic acid (EPA) and docosahexaenoic acid (DHA) from marine sources, in particular, have been studied extensively. The role of their plant-derived counterpart, alpha-linolenic acid (ALA) is less clear, yet it is the principle dietary n-3 PUFA consumed in the typical Western diet [1]. Therefore, the intent of this review is to outline the individual biological effects of ALA, EPA, and DHA, highlighting differences in their metabolism and utilization. The role of n-3 fatty acids in cancer, insulin resistance and cardiovascular disease will be reviewed, given the global prevalence of the diseases in particular and the emerging associated health benefits of the individual n-3 PUFA. Potential mechanisms by which they exert their health-related effects will also be discussed.

### Sources and Metabolism

Polyunsaturated fatty acids are hydrocarbon chains with two or more double bonds situated along the length of the carbon chain. Depending on the location of the first double bond relative to the methyl terminus, they can be classified as either n-6 or n-3. Linoleic acid (LA; 18:2n-6), the parent fatty acid of the n-6 PUFA family is an essential fatty acid and cannot be endogenously synthesized by mammals. LA is found in vegetable oils, seeds and nuts. ALA (18:3n-3), the parent fatty acid of the n-3 PUFA family, must be consumed through the diet. ALA is found in leafy vegetables, walnuts, soybeans, flaxseed, and seed and vegetable oils. Both LA and ALA can be further metabolized to long chain PUFA through a series of desaturation and elongation steps. LA is metabolized to arachidonic acid (AA, 20:4n-6), while ALA can be metabolized to EPA; (20:5n-3) and ultimately DHA (22:6n-3). Alternatively, AA can be obtained from animal fat sources and EPA and DHA can be consumed directly from marine sources.

The average per capita intake of DHA plus EPA is approximately 0.1–0.2 g per day in North America and the average per capita intake of ALA in North America is ~1.4 g daily [1]. As mentioned, ALA can be endogenously converted to EPA and DHA, however this is not an efficient process. Assessment of apparent conversion efficiency of dietary ALA to EPA and DHA is typically done by measuring the net rise in circulating EPA and DHA after increasing ALA intake. Early studies in this area found that while some moderate net rise in the level of EPA resulted with higher levels of ALA, no net rise in the level of circulating DHA occurred [2,3]. For example, feeding 10.7 g/d of ALA from flaxseed oil for 4 weeks failed to increase low DHA levels in breast milk of lactating women [4]. Some estimate that only 5–10% and 2–5% of ALA in healthy adults is converted to EPA and DHA, respectively [5], while others suggest that humans convert less than 5% of ALA to EPA or DHA [6]. The International Society for the Study of Fatty Acids and Lipids (ISSFAL) recently released an official statement on the conversion efficiency of ALA to DHA. They concluded that the conversion of ALA to DHA is on the order of 1% in infants, and considerably lower in adults [7]. Given the demonstrated benefits of DHA on visual acuity [8,9] and in the developing mammalian brain [10,11], poor conversion of ALA to DHA is a concern, particularly for vegetarians and for individuals who do not eat fatty fish.

Given the poor conversion efficiency of ALA to its longer-chain counterparts, ALA levels in the blood and tissue of humans approximate dietary intakes. Since n-3 PUFA in a typical North American diet is comprised mainly of ALA, it is pertinent to elucidate the specific effects this fatty acid. EPA and DHA intake is also low in some European countries as reviewed [12] and in India [13], making ALA the principle n-3 PUFA consumed in these regions. The prevalence of CVD [14], IR [15], and some types of cancers [16] in these countries is elevated, in contrast to countries with high fatty fish intake like Japan [17]. If conversion efficiency is the main criteria, then the epidemiological evidence above would suggest that ALA may not confer the same health benefits as its longer chain counter-parts, EPA and DHA.

### Metabolic Products of n-3 PUFA

Both ALA and LA are converted to their respective long chain metabolites by the same set of enzymes, however the metabolic products of each pathway are structurally and functionally distinct. EPA and AA are substrates for the synthesis of a group of inflammatory mediators including thromboxanes (TX), leukotrienes (LT), and prostaglandins (PG), collectively referred to as eicosanoids. Because the typical Western diet contains a much greater proportion of n-6 PUFA to n-3 PUFA, the membranes of most cells contain large quantities of AA, thus, it is typically the principle precursor for eicosanoid production [18]. AA metabolism yields 2-series PGs and 4-series LTs, highly active agents of inflammation, whereas EPA metabolism results in 3-series PGs and 5-series LTs, far less potent prostanoids by comparison [19].

Cyclooxygenase (COX) and 5-lipoxygenase (5-LOX) are enzymes required for PG and LT synthesis, respectively. Competition between n-6 and n-3 PUFA for enzymatic metabolism occurs for both PG and LT synthesis. Competition by EPA results in decreased production of TXA_2 _and LTB_4_, and PGE_2 _metabolites, which ultimately reduces platelet aggregation, vasoconstriction, and leukocyte chemotaxis and adherence [20]. In addition, metabolism of EPA gives rise to less potent eicosanoids [19]. A concurrent rise in TXA_3_, prostacyclin PGI_3_, and LTB_5 _results, inhibiting platelet aggregation and vasoconstriction and promoting vasodilation [20]. It is not difficult to associate these metabolic products and their corresponding effects with beneficial outcomes related to CVD. A decrease in platelet aggregation reduces the development of atherosclerotic plaques by making blood less viscous and decreases the likelihood of thrombus formation. Increased vasodilation promotes blood flow with reduced resistance, thus decreasing the likelihood of endothelial damage and plaque initiation.

Recent studies have identified several new groups of mediators that exert anti-inflammatory actions, via derivation from COX-2; Lipoxins (LXs) from AA, E-series resolvins from EPA [21-23] and D-series resolvins, docosatrienes and neuroprotectins from DHA [24-26]. LXs and resolvins act as anti-inflammatory mediators by assisting in the resolution of inflammatory events and assisting with the clearance of cellular debris from the site of inflammation [27]. They also suppress IL-1, IL-2, IL-6 and TNF-alpha production by T cells [28-31], thus functioning as endogenous anti-inflammatory agents. Neuroprotectin D1 possesses anti-inflammatory and neuroprotective characteristics [32,33] and has been shown to promote wound healing [34] and brain cell survival [35,36]. While this area of research requires more detailed investigation, these novel classes of inflammatory mediators may be implicated in a variety of health-related conditions.

While the conversion of ALA to its long-chain derivatives is important, human and animal studies reveal that a major metabolic fate of ALA metabolism is β-oxidation. Over a 24 hour period, 20% of palmitic, stearic, and arachidonic acids orally administered to rats were expired as CO_2_, compared to 60% for labelled ALA [37]. In humans, the values are slightly less, with 16–20% of ALA being expired as CO_2 _over 12 hours [6,38]. This corresponds with a recent tracer study in men consuming a control meal that included 700 mg of labelled ALA, which demonstrated that ~34% of the labelled ALA was recovered as CO_2 _over 24 hours [39]. In a subsequent study using test diets with elevated levels of ALA (10 g/d) or EPA+DHA (1.5 g/d) consumed for 8 weeks, it was observed that the amount of expired label in the second tracer study was not affected by increasing either ALA or EPA+DHA intakes [39]. In addition, a separate study in humans determined that ALA was the most highly oxidized fatty acid when compared to other 18 carbon fatty acids including linoleate, elaidate, oleate, and stearate [40]. Other metabolic routes of ALA include carbon recycling for de novo lipogenesis in the brain and other tissues [41].

Interestingly, whole-body ALA conversion to DHA in rats has been found to be higher than originally predicted [42,43]. In fact, in one study, the hepatic (representative of whole-body) DHA synthesis rate in rats intravenously infused with labelled ALA was approximately 30 times higher as compared to previously published rat brain DHA consumption rates [42]. Another study found hepatic DHA synthesis from ALA was only 5–10 fold higher than brain DHA consumption rates [43]. While there is discrepancy in ALA conversion rates in rats, these studies imply that dietary ALA could sufficiently supply the brain with DHA in the absence of exogenous DHA intake. It is important to note that the hepatic DHA synthesis rates observed for rats do not extend to humans [44]. The higher rates reflect a more efficient ALA elongation process in mice and rats, therefore results using these experimental models should be carefully considered when extrapolating effects in humans.

## Differential effects Of N-3 Pufa In Cancer

A growing body of literature exists surrounding n-3 PUFA and cancers of the breast and prostate. Animal studies suggest a beneficial effect, however the relationship in humans is more complex. Many human studies fail to differentiate between ALA, EPA, and DHA when reporting effects of n-3 PUFA on cancer risk, or a fish oil blend is used, preventing evaluation of individual effects of EPA and DHA. Despite these challenges, important mechanistic insights are continually being identified that will eventually help elucidate the individual effects of n-3 PUFA in two of the most common forms of cancer worldwide.

### Prostate Cancer

#### ALA

The relationship between ALA and prostate cancer has garnered considerable attention, in part due to unexpected study results reporting positive correlations between ALA intake and prostate cancer risk [45-48]. The literature is not without inconsistencies, however, as reviewed [49]. ALA intake was recently assessed by dietary questionnaire in 6 observational studies [45-48,50,51], and by blood and/or prostate tissue analysis in another 5 studies [52-56]. Of the questionnaire based studies, 4 found positive associations between ALA intake and prostate cancer risk [45-48], while no association was found in two [50,51]. In studies which measured circulating levels of ALA in blood, 2 found positive associations [55,56], while no significant relationship was established in two other studies [52,54]. In contrast, the single study that measured prostate tissue levels of ALA found a negative association between ALA status and risk of prostate cancer [53]. Two additional questionnaire based studies found no association between ALA intake and prostate cancer risk. One assessed pre-clinical prostate cancer cases [57] while the other was a nested case-control study within the Alpha-Tocopherol, Beta-Carotene cohort in Finland [58]. Interestingly, of the studies that identified positive associations between ALA and prostate cancer risk, the association was often strong and persisted or was strengthened after adjusting for potential confounding variables including total energy and fat intake, animal fat, saturated and monounsaturated fatty acids, LA, and red meat consumption. Adjusted odds ratios (OR) or relative risks (RR) varied from 1.3 to 4.3, and the association was found in populations from different countries and with diverse dietary habits, as reviewed [58,59]. One interpretation of these intriguing results could be that high levels of ALA are associated with increased prostate cancer risk because it reflects poor conversion to EPA and DHA, which have demonstrated anticancer effects.

While observational studies have offered insight into ALA and prostate cancer risk, there are inherent weaknesses associated with the study designs that limit their utility, including confounding parameters and biases relating to dietary recall and selection and classification of patients. More importantly, these observational studies do not demonstrate causality. Perhaps of more clinical relevance are the flaxseed supplementation studies recently conducted in men with prostate cancer awaiting surgery [60,61] or in men with benign prostatic epithelium [62]. These studies consistently support a protective effect of flaxseed supplementation (30 g/d for 30–180 d) by reducing cell proliferation [60-62] and increasing apoptosis [60]. Moreover, a decrease in Prostate-Specific Antigen (PSA) following flaxseed administration was observed in some of the supplementation studies [60,62]. It is difficult to draw conclusions about the effectiveness of ALA from these studies, however, as other components of flaxseed may contribute to the observed outcomes and all supplementation studies were conducted in combination with a low-fat diet. More controlled investigations of this nature are warranted, given the potential clinical utility of supplementation studies in men with prostate cancer or who are at increased prostate cancer risk due to elevated PSA levels or family history.

Animal data on ALA and prostate cancer is also limited, possibly due to inter-species diversity of anatomy, biochemistry, and pathology of the prostate gland [63]. Several studies have assessed tumorigenesis in mice, showing reductions in prostate tumour growth in mice fed EPA- and DHA-rich fish oil [64-66] but not in mice receiving ALA-rich linseed oil [64]. Similarly, ALA-rich perilla oil did not attenuate the incidence of prostate carcinoma in cancer-induced rats as compared to corn oil-fed rats [67].

Inconsistencies in the literature exist with *in vitro *investigations as well. This is complicated by the fact that experimental outcomes are derived from heterogeneous study conditions including differences in cell lines, growth conditions, and fatty acid concentrations. A number of studies have demonstrated an anti-cancer effect of ALA on prostate cancer cells *in vitro*. ALA suppressed cell proliferation and inhibited production of Urokinase-type plasminogen activator, an enzyme responsible for promoting invasion and metastasis of cancer in human DU145 cells [68]. In a separate study on the same cell line, physiological concentrations of ALA increased cell death [69]. In the PC-3 human prostatic cell line, however, ALA increased cell growth at concentrations ranging from 0.003 to 25 uM [70-73]. In contrast, EPA and DHA inhibited growth of these cells. ALA was shown to promote growth of human LN-CaP and TSU prostate cell lines, rat metastatic Mat-Ly-Lu cells, and the rat non-metastatic EPYP1 epithelial cell line [72], but had no effect on the growth of rat prostate epithelial cell lines EPYP2 and EPYP3. Overall, there is no clear association between ALA and prostate cancer in human, animal, or cell culture models and more research is warranted to clarify the effect of ALA in prostate tissue.

#### EPA and DHA

In contrast to ALA, there is some evidence suggesting a protective role for EPA and DHA in prostate cancer. *In vitro *studies have identified dose-dependent inhibition of human cancer cell growth [73] and repression of PSA [74] in PC-3, DU 145, and LNCaP prostate cancer cell lines. Further, DHA alone or in combination with a low-dose pharmacological COX-2 inhibitor (celecoxib) reduced cell growth and induced apoptosis in prostate cancer cell lines LNCaP, DU145, PC-3 and rat prostate tumour cells [75,76]. These results suggest a unique COX-2 independent mode of action of DHA+celecoxib on prostate cancer.

The seemingly protective effects of EPA+DHA observed in prostate cancer cell lines extend similarly to rodent studies. Nude mice with transplanted DU-145 human prostatic tumour cells displayed decreased tumour incidence and growth when fed diets supplemented with EPA+DHA-rich fish oil (17–20.5% w/w) [64-66]. Several studies in rodents have reported decreased prostate tumour burden with n-3 PUFA supplementation [77-79], but fail to detail the specific fatty acid composition of the n-3 PUFA in the diets, making it difficult to assess the effects of EPA and/or DHA in these investigations.

A recent review of prospective cohort studies of n-3 PUFA and prostate cancer risk in humans found that, of 7 studies evaluating risk relative to fish, marine oil or EPA or DHA consumption, 2 demonstrated either a favourable effect or a trend towards a favourable effect and the rest showed no association [80]. A significant positive association between a high LA:DHA ratio has been shown to enhance prostate cancer risk [81], eluding to a protective effect of DHA or a detrimental effect of LA on prostate carcinogenesis. The study outcomes suggest a need to take relative intakes of n-3 and n-6 PUFA into account when evaluating prostate cancer risk for a more comprehensive assessment of potential fatty acid effects. Reduced prostate cancer risk was shown to be associated with high erythrocyte phosphatidylcholine levels of both DHA and EPA [82]. In contrast, in a separate study a positive association was observed between intakes of EPA and DHA and risk of prostate cancer in initially cancer-free men aged 45–73 years [83]. While *in vitro *and rodent studies more consistently support a potentially protective effect of EPA+DHA on prostate carcinogenesis, determining the mechanisms by which they confer their benefits will be invaluable for improving our understanding in human studies.

### Breast Cancer

#### ALA

Recent observational studies have assessed breast cancer risk and breast adipose tissue fatty acid composition. Two case-control studies compared women with invasive non-metastatic breast carcinoma and women with benign breast disease [84,85]. In addition to identifying an inverse correlation between breast adipose tissue ALA and breast cancer risk, one of the studies noted a significant decrease in risk for women in the highest tertile of ALA intake [85]. Another study assessing the effects of ALA consumption on breast cancer risk reported a reduced risk for women in the highest versus lowest quintiles of ALA intake [86]. While these results are encouraging, caution must be used when interpreting data from observational studies, as correlation does not equal causation. More studies on ALA and breast cancer risk in human subjects are warranted.

In rodent models, a trend towards a protective effect of ALA on mammary tumorigenesis has been observed. A high ALA diet significantly inhibited spontaneous mammary tumorigenesis in mice [87] and feeding ALA-rich linseed oil to mice reduced growth of mammary tumours and metastasis [88]. Similar reductions in tumour growth rate and metastasis resulted when a basal diet supplemented with ALA-rich flaxseed was fed to nude mice injected with human breast cancer cells [89]. Reduced tumorigenesis was accompanied by downregulation of insulin-like growth factor I and epidermal growth factor receptor expression, offering potential mechanistic insight into the effects of ALA. Flaxseed administered to ovariectomized mice with established MCF-7 tumours demonstrated attenuation of soy protein isolate-induced tumour biomarkers after 25 weeks [90]. In a separate study in athymic mice with established MCF-7 tumours, tamoxifen in combination with a diet supplying 10% energy as flaxseed, regressed tumours to 55% of the pre-treatment tumour size [91]. Interestingly, tamoxifen alone achieved only a 6% reduction in tumour size, compared to pre-treatment values, suggesting an important anti-proliferative, pro-apoptotic role of ALA. Finally, in a study evaluating the effect of dietary beta-carotene combined with an ALA- or LA-rich diet in rats, researchers concluded that an adequate content of dietary ALA is required for a protective effect of beta-carotene in mammary carcinogenesis [92]. The results from ALA research on mammary tumorigenesis in rodents are encouraging and more work is warranted in this area to help clarify mechanisms by which individual fatty acids affect mammary gland physiology and pathology.

Few studies have investigated the effects of ALA on breast cancer *in vitro*. A study that assessed the chemoprotective potential of unsaturated fatty acids and vegetable oils observed a seemingly dual role for ALA in 17-beta-estradiol epoxidation [93]. ALA prevented formation of the potential cancer initiator 17-beta-estradiol epoxide under normal conditions. When activated by an epoxide-forming oxidant, however, ALA inhibited nuclear RNA synthesis, suggesting it might be a potential post-epoxidation carcinogen. Similarly, another study had difficulty characterizing the role of ALA in both estrogen dependent and independent breast cancer cells, citing a variable effect of ALA on cell proliferation depending on the cell line assessed [94]. ALA significantly inhibited cell growth in ER-negative MDA-MB-231 and HBL-100 human breast tumour cells but not in ER-positive MCF-7 cells. A trend towards a decrease in cell growth in the other ER-positive cell lines ZR-75 and T-47-D by ALA did not reach statistical significance [94]. Authors did identify, however, that the addition of ALA, EPA and DHA to breast cancer cells increased the content of conjugated dienes and lipid hydroperoxides in cellular lipids, which was significantly correlated with the capacity to arrest cell growth.

#### EPA and DHA

The data for EPA and DHA in breast cancer are equivocal. Some case-control studies have demonstrated significant inverse associations between breast cancer risk and dietary intake of n-3 PUFA from fish and fish oils. Bagga et al. showed a decreased risk of breast cancer development with higher EPA and DHA consumption [95]. Similarly, an investigation assessing erythrocyte n-3 PUFA levels from fish consumption identified an inverse association with breast cancer risk [17] and another assessment of erythrocyte fatty acid composition found the inverse association significant only for EPA and total n-3 PUFA content [96]. Contrary to these findings, however, a large study of post-menopausal women concluded that increased fish consumption and thus, increased EPA and DHA intake, was associated with elevated breast cancer rates, but only in ER+ breast cancers [97]. Others assessing fish consumption and breast cancer have found no significant associations [98,99].

EPA and DHA have demonstrated protective effects in a number of rodent models of breast cancer. Fish oil supplementation decreased tumour growth rates and the extent of metastases in BALB/cAnN and nude mice [100,101]. Similarly, supplementing in nude mice with EPA and DHA independently produced comparable results [102]. Chemically-induced mammary tumorigenesis has been studied in rats with similar outcomes. Corn oil increased growth of DMBA-induced mammary tumours, while menhaden oil inhibited their development at corresponding supplementation levels [103]. In a separate investigation, menhaden oil at 20% of energy reduced tumour incidence and prolonged tumour latency, with authors determining that EPA was significantly inversely related to mammary tumour development [104]. DHA has also been shown to decrease mammary tumour incidence [105], yielding a 60% increase in BRCA1 protein level, the product of a major tumour suppressor gene. Fish oil supplementation has also been shown to enhance the therapeutic effects of tumour inhibitors doxorubicin and mitomycin C in mice [106,107].

Cell culture studies also support the protective role of EPA and DHA in breast cancer. Anti-proliferative effects have been observed for both EPA and DHA in human mammary epithelial cells, with a higher efficiency noted for DHA [108]. Moreover, both EPA and DHA inhibit MCF-7 cell growth by 30 and 54%, respectively [109], and they have decreased FAS activity [110], a possible oncogene that is up-regulated in breast cancers [111]. A study of BT-474 and SkBr-3 breast cancer cells, which naturally amplify the HER-2 oncogene, found that DHA downregulated HER-2 action [112]. Another *in vitro *investigation identified dose-dependant cytotoxic effects of EPA and DHA on human breast tumour cells [113] and arrested tumour cell growth in numerous estrogen-dependent and -independent cell lines [94]. The results from *in vitro *and rodent studies support a protective effect of EPA+DHA on mammary tumourigenesis, however a clear definition of their role in human breast cancer is still lacking, which requires additional mechanistically focused studies.

### Cancer-Specific Mechanisms of n-3 PUFA

#### ALA

ALA, mainly as a component of flaxseed, has been shown to decrease angiogenesis and metastasis in some studies [114,115], but not others [116]. *In vitro*, Menendez et al. studied breast cancer cells naturally amplifying the HER-2 oncogene and found that ALA suppressed HER-2 coded p185 Her-2/neu oncogene expression [117]. While the precise mechanism responsible for the suppression is unknown, it was determined to have occurred at the transcriptional level, suggesting a fundamental change in RNA synthesis. Further, dose-dependant cytotoxic capabilities of ALA on human breast tumour cells have been identified [113], offering potential ways in which this fatty acid might be anti-carcinogenic.

#### EPA and DHA

In addition to the anti-inflammatory mechanisms described previously, EPA-derived products of COX and LOX decrease tumour growth [118,119] and EPA and DHA individually decrease activation of oncogenic transcription factors [120,121]. They inhibit angiogenesis [122-126], downregulate expression of Bcl-2 family genes [127,128], and promote apoptosis by downregulating NF-kB [129]. DHA has also been shown to halt tumour growth by promoting differentiation of breast cancer cells [130], which prevents further cell multiplication. Further, EPA and DHA incorporation into membrane rafts (MRs) reduces total cholesterol content and ultimately enhances apoptosis in epithelial, prostate and cancer cells via Akt inactivation [131]. Antiproliferative action and apoptosis has also been demonstrated by EPA and DHA through inhibition of HMG-CoA reductase [132], which inhibits the mevalonate pathway and, ultimately, the function of oncogenic forms of Ras.

## Differential effects of N-3 Pufa in Insulin Resistance

Insulin resistance plays a role in several chronic diseases including metabolic syndrome and type 2 diabetes (T2D). There is a growing body of evidence suggesting an inverse association between n-3 PUFA and insulin resistance (IR). Anti-diabetic effects of PUFA have been observed, including increased basal metabolic rate and fat oxidation [133,134], however some of these findings have resulted from studies comparing polyunsaturated:saturated fatty acid intake. While identifying differences in energy substrate utilization based on the saturation ratio of dietary fatty acids is important, it is of interest to determine any fatty acid-specific differences that might exist among n-3 PUFA.

### ALA

To date, few studies have examined the impact of ALA consumption on markers of T2D and IR. In one investigation, T2D subjects received safflower oil or 60 mg/kg body weight/day flaxseed oil, translating to roughly 5.5 g ALA/day. After 3 months of supplementation, no significant changes were observed in fasting blood glucose, insulin, or HbA1c [135]. In a separate study, the inflammatory marker C-reactive protein (CRP), but not IR, was inversely related to blood plasma phospholipid and cholesteryl ester levels of ALA, as well as EPA and DHA in persons with metabolic syndrome [136]. Two additional studies failed to note any significant change in insulin, and glucose after supplementing T2D subjects with 35 mg/kg body weight ALA in the form of flaxseed oil for 3 months [137,138]. In contrast, Enriquez et al. observed a positive correlation between fasting insulin levels, IR, and erythrocyte ALA content in a comparable T2D population [139]. Based on the limited data available, no conclusions can be made regarding ALA and markers of T2D, although preliminary evidence does not seem to support an insulin-sensitizing role of ALA in T2D. Comparable studies on healthy individuals would be useful to identify any potential beneficial preventative effects of ALA on IR or T2D.

To the best of our knowledge there are no cell culture studies investigating the effect of ALA on IR. Only a few rodent studies have reported effects of ALA. Recently, Javadi et al. assessed the effects of 12% w/w ALA:4% w/w LA on body composition in mice. After 35 days, the proportion of body fat was not influenced by increased dietary ALA:LA, as compared to high LA:ALA or low-fat diets [140]. Plasma total cholesterol and phospholipids were significantly lower in the high ALA compared to the high LA group and the activities of enzymes in the fatty acid oxidation pathway were significantly raised in both PUFA groups vs. the low-fat diet group. There were, however no differences in fatty acid oxidation or lipogenic enzymes between the high ALA and LA group, indicating no significant influence of ALA on body composition. In contrast, Ghafoorunissa et al. demonstrated that substituting one third dietary LA with ALA significantly improved insulin sensitivity and decreased blood lipid levels in sucrose-induced IR rats [141]. Similarly, ALA-rich chia seed prevented the onset of dyslipidaemia and IR in rats fed a sucrose-rich diet for 3 weeks [142]. Further, dyslipidaemia and IR in rats receiving a sucrose-rich diet for 3 months were normalised and visceral adiposity was reduced when they were fed chia seed for the last 2 study months. While the extent to which ALA, specifically, was responsible for the beneficial effects seen in the chia seed group is unknown, the results are encouraging and warrant further investigation. ALA has also significantly improved insulin sensitivity and glycemic response in male ob/ob mice [143].

At present, there are too few studies on ALA in this area of research to delineate its effects. Further, animal studies have used varying ALA concentrations and in varying ratios with LA, making it difficult to accurately define a role for ALA, specifically. As well, use of high levels of ALA in rodent studies should be cautiously interpreted, as they may not be physiologically relevant in humans. It can be hypothesized that, at high enough concentrations, ALA could be converted into levels of EPA and DHA that reach therapeutic levels, particularly given the current discrepancy regarding the efficiency of ALA conversion to its longer chain derivatives in rats [42,43]. Apart from its ability to convert to EPA and DHA, however, it would be of value to elucidate any specific bioactive effects ALA might have in relation to T2D and its related pathologies. Recently developed mouse models, including a delta-6-knockout mouse that inhibits the conversion of ALA to EPA and therefore DHA [144], could be highly useful in this regard.

### EPA and DHA

Findings involving fish oil effects on human body composition and IR vary depending on the health of the subjects and the nature of the study. As a result, it has been difficult to determine the effects of EPA+DHA on diabetes-related parameters. Body fat mass decreased and lipid oxidation was concurrently stimulated in healthy volunteers when 6 g/d visible fat was substituted with 6 g/d fish oil [133]. Browning et al. recently reported that after 12 weeks of EPA and DHA supplementation in overweight women, a significant reduction in inflammatory markers was observed [145], although it was unclear whether the seemingly insulin-sensitizing effects of n-3 PUFA were mediated through inflammatory mechanisms. Another study, however, did not identify any correlation between dietary intakes of EPA and DHA and IR in T2D subjects, as measured by HOMA-R [146]. It appears as though PUFA from marine sources potentially contribute to favourable modifications of diabetes-related parameters, possibly by increasing insulin sensitivity, decreasing inflammatory mediators, or altering lipid metabolism in lean adults. This benefit, however, does not seem to extend to obese or T2D subjects.

Animal studies involving EPA+DHA and IR tend to be more consistent and support an anti-diabetic effect. Numerous rodent studies have shown that EPA improves IR in several models of obesity and diabetes [147-149] and elevated systemic concentrations of insulin-sensitizing adiponectin [150] as well as an improved response to a glucose load [151] were reported in mice fed high fat diets enriched in EPA+DHA. Several studies have assessed fish oil feeding in sucrose-fed rats and noted attenuated peripheral IR, hyperglycemia, and fat pad mass [152,153] as well as increased insulin-stimulated glucose transport [154] in supplemented animals. EPA as well as DHA prevented alloxan-induced diabetes and restored the anti-oxidant status of various tissues to normal range in rats [155] and were shown to be more effective than ALA at lowering plasma glucose and insulin levels and improving insulin sensitivity [156]. When a 60% energy from fructose diet was supplemented with 4.4% energy from fish oil, the hyperlipidemia that occurred in unsupplemented rats was prevented, however hyperinsulinemia was not [157]. The findings of a study on male ob/ob mice, however, concluded that EPA+DHA had no effect on insulin sensitivity or fasting blood glucose [158].

Many *in vitro *studies assessing IR have cultured adipocytes from insulin resistant and insulin sensitive rodents that have been fed diets differing in EPA+DHA content. Several of these studies demonstrate improved insulin-stimulated glucose transport, oxidation, and incorporation into total lipids in the adipocytes of normoinsulinaemic rats fed a sucrose-rich diet including 30% of energy as fish oil [159,160]. Similarly, rats fed a sucrose-rich diet long-term for 120 d were hypertriglyceridemic, insulin resistant, and had abnormal glucose homeostasis [153]. When 7% w/w fish oil was isocalorically substituted for corn oil from day 90–120, the inhibitory effect of the high-sucrose diet on the antilipolytic action of insulin was corrected and the *in vitro*-enhanced basal lipolysis was reduced [153]. An investigation by Baker and Gibbons also supports a favourable role of dietary fish oil with respect to IR in rat hepatocyte cultures [161]. The hepatocytes from rats fed an 18% w/w fish oil diet for 2 weeks demonstrated significantly altered sensitivity of insulin to some aspects of *in vitro *hepatic fatty acid and glycerolipid metabolism [161]. Compared to hepatocytes from rats fed a low-fat or olive oil-containing diet, fish oil feeding abolished the inhibitory effect of insulin on the oxidation of exogenous oleate. Compared to the olive oil and low-fat groups, however, the fish oil-fed group had little to no effect on insulin's ability to stimulate the incorporation of oleate into triglycerides (TG). There was also no change in the sensitivity of VLDL TG secretion to inhibition by insulin in the fish oil group [161]. Thus, dietary supplementation with fish oil might differentially affect the metabolic pathways of the liver, however until more research is done, it will not be clear exactly how EPA+DHA are implicated mechanistically in IR.

### IR-Specific Mechanisms of n-3 PUFA

n-3 PUFA are proposed to reduce the risk of insulin resistance in multiple ways, few of which seem to be differentially affected by the 3 fatty acids.

#### ALA

While there are no clear lipid-specific mechanisms by which ALA might affect insulin resistance, one investigation assessing the effects of ALA *in vitro *and *in vivo *suggests a potential anti-oxidant, anti-cytotoxic role of this fatty acid. Prior exposure of an insulin-secreting rat insulinoma cell line to ALA in culture was shown to prevent alloxan-induced cytotoxicity and apoptosis [155]. In the same study, prior supplementation with ALA also prevented alloxan-induced diabetes in live rats and restored anti-oxidant status to normal range in various tissues. The anti-oxidant action of ALA is encouraging, as oxidant stress is typically elevated in diabetics. The following effects noted for EPA and DHA also extend to ALA, including upregulation of insulin receptors and PPARs and downregulation of NF-kB. The impact of EPA and DHA on these parameters, however, tends to be more potent.

#### EPA and DHA

EPA and DHA are preferentially incorporated into cell membranes, thus increasing membrane fluidity. This, in turn, has been shown to increase the number of insulin receptors on the cell membrane and their affinity to insulin [162]. Upregulating insulin receptors decreases insulin resistance and favourably modifies an individual's glycemic response, an effect that could potentially delay or prevent onset of T2D. Transcription factors have also been implicated in IR. NF-kB activation of endothelial cells has been demonstrated in response to hyperglycemia, however EPA and DHA have been shown to downregulate NF-kB [163]. This could potentially mediate some of the vascular complications that result from chronically elevated glucose levels seen in diabetics. Further, PPARγ has been implicated in the etiology of IR, as it increases the expression and translocation of GLUT-1 and GLUT-4, thereby facilitating glucose uptake in adipocytes and muscle cells [164]. EPA and DHA act as ligands for PPARs and thus, may have an anti-diabetic role. Moreover, stimulation of PPARγ inhibits expression of IR-promoting cytokines, while concurrently triggering an increase in plasma concentrations of adiponectin [165]. This has the net result of decreasing blood levels of glucose by improving insulin sensitivity and decreasing liver glucose production [166].

## Differential effects of N-3 Pufa in Cardiovascular Disease

Perhaps the most robust evidence for potentially beneficial effects of EPA and DHA has resulted from research surrounding cardiovascular health [167-171]. In contrast, a clear relationship between cardiovascular disease (CVD) and ALA intake in humans is lacking.

### ALA

In an attempt to determine potential differential effects of n-3 PUFA, Singh et al. compared the effects of feeding ALA-rich mustard seed oil, fish oil, and a non-oil placebo to 360 patients hospitalized for suspected acute myocardial infarction (MI) [172]. They found that both oil supplements reduced CVD outcomes, including total cardiac events and non-fatal infarctions, but only the effects of the fish oil reached statistical significance. Further, fish oil but not mustard seed oil reduced the number of total cardiac deaths reported [172]. Natvig et al. randomly assigned 13,578 healthy subjects to receive 10 ml flaxseed oil (5.5 g ALA) or 10 ml sunflower seed oil (0.14 g ALA) daily for a year and observed no significant cardiovascular benefit of ALA supplementation [173]. Conversely, several studies assessing the effects of ALA intakes of between 1.8 and 6.3 g/d [174-176] reported significant reductions or trends toward reduced numbers of CVD events [174-176]. The validity of some trials mentioned here [172,175] has been questioned by reviewers, citing multiple methodological issues such as inadequate randomization concealment, the use of a non-oil placebo, and even calculation errors in the published results [177,178]. Accordingly, assertions cannot be confidently made regarding the potential of ALA to have cardioprotective effects, despite some intriguing study findings.

A recent meta-analysis was conducted to determine whether ALA supplementation could modify 32 established and emerging cardiovascular risk markers [179]. Of the 14 studies reviewed, only 3 outcomes – fibrinogen, fasting blood glucose, and HDL cholesterol – were modified by at least 4 weeks of ALA supplementation, prompting authors to conclude that ALA supplementation to reduce CVD could not be recommended [179]. In contrast, a meta-analysis of 5 prospective cohort studies and 3 clinical trials assessing ALA intake and risk of fatal coronary heart disease concluded that ALA intake might reduce heart disease mortality [180].

Several independent analyses of the NHLBI Family Heart Study have identified multiple inverse associations between ALA and CVD risk factors including prevalence of hypertension, coronary artery disease, plasma TG, and carotid atherosclerosis [181-184]. Some studies have demonstrated cardioprotective effects of ALA on risk of MI [176,185-187], stroke [188], and ischemic heart disease [189]. Others have found no significant association between MI and ALA [190]. The inconsistencies in these study results is not entirely unexpected, however, as there is significant heterogeneity in the study populations and designs. In addition, several of the studies assessed nutrient intake by dietary questionnaire, which can yield errors in food intake estimates and nutrient content calculations of specific foods. Moreover, background EPA, DHA and/or fish consumption might mask the effects of ALA intake [186], offering a potential explanation as to why some researchers have found no associations between nonfatal MI and ALA intake.

### EPA and DHA

Mounting evidence from epidemiological and dietary intervention trials supports the cardioprotective role of EPA+DHA-rich fish oil [167-171]. Their demonstrated beneficial effects include, but are not limited to regulation of eicosanoid production from AA, plasma triacylglycerol- and blood pressure-lowering effects, regulation of ion flux in cardiac cells, and regulation of gene expression via the peroxisomal proliferation system, as reviewed by Sinclair, et al. [191]. It is well-known that EPA+DHA favourably modify serum markers of CVD risk by reducing TGs and increasing HDL-cholesterol and there was a meta-analysis on this topic in 2006 [192]. In particular, their TG-lowering ability has been demonstrated at intakes that are achievable from the diet [167-169,171], providing compelling evidence for effective dietary CVD therapy.

While the majority of investigations assessing CVD and fatty acid intake suggest a beneficial effect of marine-derived PUFA, Burr et al. reported that fish oil supplements but not fish intake increased the incidence of sudden cardiac death in patients with angina [193]. However, as recently summarized, the research collectively shows beneficial effects of n-3 PUFA from both marine and plant sources on sudden cardiac death incidence [170]. Despite the study by Burr and colleagues, the effectiveness of n-3 PUFA as an agent for the secondary prevention of cardiovascular events seems promising, following a recent review of 4 secondary prevention trials [194]. All 4 trials reduced secondary cardiac events with between 1.0 and 1.8 g/d fish oil capsules or with 1 serving of fish/d or ALA supplementation. Further, results were similar irrespective of form of n-3 PUFA intake, providing a practical and attractive option for widespread CVD therapy. The cardioprotective effects of EPA and DHA from marine sources are well documented and offer a promising avenue by which North Americans can reduce their risk of CVD through dietary means.

### CVD-Specific Mechanisms of n-3 PUFA

Perhaps the most robust evidence for the health-promoting effects of fatty acids is derived from studies assessing the relationship between n-3 PUFA and CVD [167-171]. As a result, much work has been done in this area and, increasingly, a focus on differentiating between the effects of ALA and EPA+DHA is occurring.

#### ALA

Some have speculated that the seemingly protective effects of ALA may have more to do with cardiac function than with plasma lipids [5]. While ALA supplementation has decreased total cholesterol, effects have been minimal (2 or 8% reduction from baseline at 3.5 or 5.3% energy as ALA, respectively) [195,196]. ALA has, however, significantly reduced the incidence of arrhythmias and cardiac mortality in rats [197], enhanced arterial compliance in obese subjects [198,198], and decreased C-reactive protein, IL-6, and serum amyloid A – inflammatory markers implicated in atherogenesis in males with dyslipidaemia [199]. While effects of ALA on platelet aggregation and thrombosis are inconsistent [200], there seems to be an overall protective effect of this fatty acid on cardiac outcomes in humans and rodents that is not explained solely by modest reductions in cholesterol levels.

#### EPA and DHA

EPA and DHA are potent hypotriacylglycerolaemic agents. Analysis of 36 human crossover studies found 3–4 g/d EPA+DHA intake yielded a plasma TG decrease of 24% and 35% in normolipidaemic and hypertriacylglycerolaemic subjects, respectively [201]. This is thought to be due to both decreased TG synthesis, likely via impairment of the SREBP pathway [202], and increased TG clearance by EPA+DHA. N-3 PUFA from marine sources have also demonstrated antiarrhythmic effects. At 2.4 g/d, EPA+DHA significantly reduced ventricular premature complexes in patients with frequent ventricular arrhythmia and at 4 g/d EPA+DHA, heart rate variability was increased in survivors of MI, as reviewed by Wijendran et al. [5]. Fish oil has improved arterial compliance and endothelial function [203] and decreased blood pressure in a dose-dependant manner [204]. Further, DHA but not EPA significantly improved forearm blood flow and vascular reactivity in hyperlipidaemic, overweight men [205]. Apart from these antiatherogenic properties, EPA+DHA have demonstrated antithrombotic action, however not at clinically relevant supplementation intake levels [5].

## Potential global mechanisms of action

Currently, proposed mechanisms of how n-3 PUFA impact physiological processes include: regulation of inflammation, alteration of gene expression, modification of membrane raft structure and function, and involvement in other disease-specific pathways.

### Membrane effects of n-3 PUFA

N-3 and n-6 PUFA compete not only for the same set of metabolic enzymes, but also for incorporation into cell membranes, where they influence membrane fluidity and the function of membrane-bound constituents, including receptors and enzymes. ALA, EPA, and DHA differentially impart fluidity in cell membranes, however the individual n-3 PUFA effects have not been studied equally in this area. The identification of abundant amounts of DHA in the retina and brain has led to a greater proportion of research on this fatty acid compared to ALA and EPA. As a result, the effects of ALA and EPA in membranes are not entirely clear.

#### ALA

In humans, increased membrane fluidity results following ALA supplementation. At 0.9% of total energy, ALA increased erythrocyte membrane fluidity in 29 supplemented monks [206], particularly when intake of myristic acid, a saturated fatty acid, was reduced. Fluidity was measured by labelling red blood cells with 16-doxylstearate and subsequently calculating relaxation-correlation time. ALA membrane enrichment has also been demonstrated *in vitro *with various outcomes depending on the cell line studied [207-209]. An important role for ALA in skin and fur has also been investigated following the observation that ALA (and LA) supplementation reduced skin lesions in rats [210]. Subsequently, ALA enrichment in skin and secretion onto fur has been noted in guinea pigs [211], rats [212,213], and primates [214]. Proposed roles for this fatty acid are to promote fur growth and to offer protection of fur and skin from damage by sun, water, and other agents, as reviewed by Sinclair et al. [191].

#### EPA

EPA has demonstrated notable membrane modification in immune cells. Immune cells are typically rich in AA, which produces pro-inflammatory eicosanoids. Immune cell fatty acid content can be modified, however, through oral administration of EPA and DHA, which displaces AA from the membranes [18]. EPA, specifically, has been shown to inhibit AA release from phospholipids by phospholipase A2 [215], effectively reducing the amount of substrate available for the production of potent pro-inflammatory eicosanoids. Altering immune cell fatty acid composition can also influence phagocytosis, T-cell signalling, and antigen presentation capability, effects which are likely mediated at the membrane level. While several beneficial effects of ALA and DHA also have an anti-inflammatory or immune component, EPA seems to be particularly potent at decreasing inflammation. EPA may also have an important role in bone development and remodelling [216-220] and has been implicated in myelin sheath membrane maintenance and stabilization [221], as well as attenuating protein degradation in skeletal muscle of cachectic cancer patients [222-224]. These EPA-specific effects, however, will not be covered in this review.

#### DHA

DHA is a key player in conferring fluidity to rhodopsin disks in rod cells of the eye [225] and axons in the mammalian brain [226]. DHA has demonstrated an ability to alter phase behaviour in cell membranes by distorting packing by steric restrictions associated with the presence of multiple rigid double bonds, which decreases membrane stability [227]. There have also been numerous reports linking DHA to increased membrane permeability and a predisposition to undergo membrane vesicle formation and fusion [228]. These traits are proposed to be due, in part, to looser lipid packing conferred by DHA in membranes, which would facilitate deeper penetration of water and other solutes in the bilayer and that acyl chain unsaturation and membrane curvature combine to favour fusion [227]. EPA also increases plasma membrane fluidity of cells, but has been shown to accomplish this to a lesser extent than DHA [229]. This is thought to be due to its slightly shorter chain length and thus, reduced ability to decrease membrane cholesterol content and increase the unsaturation index in the plasma membranes.

In addition, a 'membrane pacemaker theory' has recently been proposed, in which DHA-enriched membranes are associated with high metabolic rates of tissues such as heart and skeletal muscles [230,231]. The theory seems to be successful at correlating n-3 PUFA status with metabolic rates notably that, as membrane content of DHA increases and the degree of polyunsaturation increases, a corresponding increase in the activity of membrane-associated processes is observed [232]. It has been proposed that such membrane polyunsaturation increases the molecular activity of many membrane-associated proteins and consequently some specific membrane leak-pump cycles and cellular metabolic activity.

### Membrane Rafts

Lipid rafts, also termed lipid microdomains, detergent-resistant membranes (DRMs), Triton-X insoluble membranes, and membrane rafts (MR), are distinct plasma membrane regions ~100 nm – 200 nm in diameter [233] with reduced fluidity due to their enrichment in cholesterol, glycosphingolipids, and phospholipids [234-236]. Caveolae, viewed as a subset of lipid rafts, are ~100 nm diameter flask-shaped invaginations of the plasma membrane, rich in cholesterol, glycosphingolipids, and the cholesterol-binding protein caveolin [236,237]. Caveolae involvement have been identified in studies on cancer [238-241], IR [242-245], and CVD [246-250] and the caveolae-specific protein caveolin is being implicated in numerous signalling pathways as our understanding of caveolae and its constituents expands.

Membrane rafts are thought to be key elements in signal transduction, ion channel function, trafficking, and protein sorting [251-255] and are the target of many acylated proteins [256-258]. The precise role rafts play, however, remains to be determined. Similarly, the individual effects of ALA on membrane raft structure and function requires investigation. Indeed, there are currently no studies of this nature. Experimentation in this area is necessary to identify mechanisms involved and pathways affected by dietary intake of ALA, and how they compare to those of its longer-chain counterparts.

Alterations in dietary EPA and DHA intake modify lipid raft structure due to their highly unsaturated nature and inability to pack efficiently with the highly saturated acyl side chains present in MRs. This, in turn, has resulted in altered lipid raft function [259-261]. N-3 PUFA enrichment of MRs has been demonstrated in mammary, colon, epithelial, and prostate cells, affecting various signalling pathways depending on the cell line involved [262,263].

EPA was recently shown to profoundly alter lipid composition and fatty acyl substitutions of phospholipids in caveolae [264]. In the same study, investigators identified EPA-induced translocation of eNOS from caveolae to soluble fractions, accompanied by displacement of caveolin from caveolae. In contrast, Bousserouel et al. concluded that EPA (and DHA) treatment increased caveolin concentration in caveolae, which correlated with smooth muscle cell proliferation inhibition [265].

DHA has demonstrated an ability to alter lipid raft size and distribution [266] and behaviour [227,267]. DHA treatment markedly altered the lipid environment of caveolae in endothelial cells, resulting in selective displacement of caveolin and eNOS [268], and inhibited cytokine production and signalling [89,269], suggesting a role for DHA-induced modifications of caveolae in atherosclerosis and other inflammatory conditions.

### Inflammation

It is widely accepted that a chronically upregulated inflammatory state is involved in the etiology of cancer, IR, and CVD. As detailed previously, when n-3 PUFA intake increases, a corresponding increase in AA antagonism occurs and the production of less inflammatory and chemotactic derivatives results, decreasing an individual's susceptibility to developing chronic inflammatory problems and related diseases.

#### ALA

Adhesion molecules including intracellular adhesion molecule-1 (ICAM-1), vascular adhesion molecule-1 (VCAM-1), and E-selectin, upon upregulation, facilitate the movement of immune cells into tissue and promote inflammation. ALA has been shown to reduce plasma concentrations of soluble E-selectin and VCAM-1 in healthy human subjects [270]. Epidemiological studies have further demonstrated reduced plasma concentrations of markers of inflammation including C-reactive protein and IL-1ra [271], as well as IL-6 and E-selectin by ALA (at ~0.6 g/d) [272]. Intervention trials found similar anti-inflammatory effects, although results were obtained with high ALA intakes (5–15 g/d) [199,270,273-275]. Reductions in C-reactive protein and the adhesion molecules and pro-inflammatory cytokines mentioned above have been associated with reduced risk of CVD [276], suggesting a potential mechanism of action for ALA in cardiovascular health promotion.

#### EPA and DHA

Apart from altered eicosanoid production discussed previously, EPA inhibits IL-2 production by peripheral blood mononuclear cells of some human donors [277] and both EPA and DHA can inhibit IL-1*B *and TNFα production by monocytes [278] and the generation of IL-6 and IL-8 by venous endothelial cells [279,280]. Overproduction of these cytokines can be dangerous, as they are implicated in the pathological responses that occur in inflammatory conditions. In addition, DHA decreased the surface expression of multiple cell adhesion molecules on ex vivo human venous endothelial cells [281] and impaired the adherence of ligand-bearing monocytes [282].

### Gene Expression

A more direct target proposed for n-3 PUFA is regulation of the expression of genes involved in inflammation. ALA, EPA and DHA have all demonstrated reduced cytokine-mediated induction of expression of inflammatory genes in culture [283]. The downregulation of inflammatory gene expression has been proposed to be mediated through nuclear factor kappa B (NF-kB) and peroxisome proliferator-activated receptors (PPARs). NF-kB, in its inactive form, has an inhibitory subunit (IkB) that, upon stimulation, is phosphorylated and dissociates from the rest of the inactive NF-kB heterotrimer. The remaining NF-kB unit translocates to the nucleus and regulates the transcription of target genes.

Unlike NF-kB, PPARs dimerise with retinoid-X-receptors (RXRs) to regulate gene expression. PPAR-alpha and -gamma are found in inflammatory cells and play important roles in the liver and adipose tissue, respectively. They are thought to be regulated, in part, by direct binding of PUFA and eicosanoids and have been proposed to stimulate inflammatory eicosanoid degradation via induction of peroxisomal B-oxidation. Alternatively, PPARs might interfere with activation of other transcription factors, including NF-kB, as previously reviewed [18].

ALA has demonstrated anti-inflammatory effects via NF-kB suppression in multiple cell lines *in vitro *[284-287] and of 10 different fatty acids (excluding EPA and DHA) tested for their bioactivites on PPAR-gamma, ALA was determined to be the most potent activator [288].

The inhibitory effects of EPA and DHA on NF-kB have recently been reviewed [289]. EPA and DHA administration in fish oil has also reduced mRNA levels of inflammatory mediators including TNF-alpha, IL-1B, and IL-6 in various animal studies [290-292], confirming a mechanistic link between inflammation, EPA+DHA, and gene expression. A connection has also been identified between EPA and DHA and the function, distribution, and activation of PPARs, given their antagonistic effect on LTB_4 _production and action [293]. This suggests an influential role of n-3 PUFA on PPARs, which has been supported by others [294]. EPA and DHA have also been shown to be more potent *in vivo *activators of PPARα than other fatty acids [295], suggesting a preferential role of these fatty acids in PPAR pathways.

## Limitations and considerations

While research on n-3 PUFA has produced exciting results, it is not without inconsistencies and there are several factors that currently limit the utility of some study outcomes. For example, food frequency questionnaires, often used in nutritional epidemiology as a method of assessing dietary intake, may produce inaccurate results. Questionnaires are subject to recall bias and the food composition databases they are based upon may lack precision in quantifying actual nutrient intake. Alternatively, erythrocytes have been used as biomarkers for dietary intake of fatty acids, however, they too lack complete accuracy. Some sources indicate erythrocyte membrane fatty acid composition is reflective of typical diet at approximately 4 months [296], whereas other research suggests RBC membrane levels of fatty acids reflect dietary intake after only 3 weeks [96,297]. Further, fatty acid levels in the blood do not necessarily accurately predict levels in all tissues, possibly due to inter-individual differences in fatty acid metabolism [298]. Identification of tissue-specific biomarkers for fatty acid intake would be of high utility.

The relationship between ALA and chronic disease is unclear. In terms of research on insulin resistance, cell culture work is lacking, however animal studies tend to support a beneficial role of ALA on insulin sensitivity. On the other hand, human outcomes demonstrate a greater degree of variability. This could be explained, in part, by the fact that supplementation study results can be confounded by background intake of fish, walnuts, flaxseed, or other n-3 PUFA-rich foods in humans [177,186].

Similarly, research on ALA and prostate cancer in rodents fails to demonstrate any significant association, while human dietary questionnaire-based studies suggest a trend towards a tumour-promoting role of ALA. Interestingly, blood and tissue analyses in this area produce a wide range of results, from positive associations between tissue ALA and prostate cancer to negligible or negative associations between ALA levels in the blood and prostate cancer risk. In contrast, the literature surrounding breast cancer and ALA is more consistent and suggests an anti-tumourigenic effect of this fatty acid in rodents and humans. Several factors could be contributing to such variability in study results, including tissue-dependent differences in tumorigenesis, diverse modes of ALA supplementation and measurement, and variability in study length, subject characteristics and outcome measures. Further, ALA-rich flaxseed, which is often used in human supplementation studies, has varying degrees of bioavailability depending on whether it is administered in its whole, ground, or oil form [299].

The robust cardioprotective effects of n-3 PUFA from marine sources are well documented, however a general consensus on the beneficial relationship between ALA and CVD is still lacking. Part of the problem stems from the fact that chronic diseases like CVD take many years to develop and are often defined by the co-existence of multiple risk factors. Further, each risk factor could be differentially impacted by ALA and other dietary fatty acids making it difficult to determine the precise mechanisms and complex interrelationships involved. This could help account for some of the discrepancy in the literature surrounding ALA and CVD. The results of several recent human studies, however, are intriguing and warrant further investigation.

## Future directions and conclusion

Research has assessed the effects of n-3 PUFA in diverse models of disease with different study designs and varying outcome measurements. While this is a valuable contribution to the breadth of the literature, additional mechanistic and human studies are warranted to further substantiate previous findings. There is growing recognition of the potential heterogeneous effects of ALA, EPA and DHA, which should be considered in future experimental designs.

Clarification of the relationship between n-3 PUFA and cancer at multiple time points is also needed. Typically, cancer studies are conducted in older individuals who have already naturally accumulated considerable DNA damage, or who have existing tumours or malignancies. The potential preventative contribution of ALA, EPA and DHA during mammary or prostate gland development, however, has yet to be detailed. This could help identify fatty acid effects at critical developmental time points that could modify future breast and prostate health.

It is also necessary to clarify how the mode of n-3 PUFA administration affects physiological outcomes. N-3 PUFA can be obtained from either dietary sources or via supplementation, and inherent challenges exist with both options when attempting to determine resultant n-3 PUFA-specific effects. To further advance the field of PUFA research, it would be useful for future studies to tease out the effects of dietary n-3 PUFA from the matrix of food, which has additional biologically active components.

The health-related effects of EPA and DHA have undergone considerable study, however the specific biological effects of ALA are largely unknown. Therefore, more work is required to identify the differential effects of ALA on cancer, insulin resistance and cardiovascular disease. The need is evermore apparent, given that ALA is by far the predominant form of n-3 PUFA consumed in the typical North American diet and its conversion to EPA and DHA is minimal. Identification of potentially beneficial or detrimental effects of ALA intake thus may have a profound and widespread impact on health promotion or disease burden.

## Competing interests

The authors declare that they have no competing interests.

## Authors' contributions

BM Anderson was the primary author. DWL Ma provided assistance in the writing and editing of the manuscript. All authors read and approved the final manuscript.
